# Power Allocation and Energy Cooperation for UAV-Enabled MmWave Networks: A Multi-Agent Deep Reinforcement Learning Approach

**DOI:** 10.3390/s22010270

**Published:** 2021-12-30

**Authors:** Mari Carmen Domingo

**Affiliations:** Department of Network Engineering, BarcelonaTech (UPC) University, 08860 Castelldefels, Spain; cdomingo@entel.upc.edu

**Keywords:** Unmanned Aerial Vehicles (UAVs), energy harvesting, energy cooperation, power allocation, Multi-Agent Deep Reinforcement Learning (MADDPG)

## Abstract

Unmanned Aerial Vehicle (UAV)-assisted cellular networks over the millimeter-wave (mmWave) frequency band can meet the requirements of a high data rate and flexible coverage in next-generation communication networks. However, higher propagation loss and the use of a large number of antennas in mmWave networks give rise to high energy consumption and UAVs are constrained by their low-capacity onboard battery. Energy harvesting (EH) is a viable solution to reduce the energy cost of UAV-enabled mmWave networks. However, the random nature of renewable energy makes it challenging to maintain robust connectivity in UAV-assisted terrestrial cellular networks. Energy cooperation allows UAVs to send their excessive energy to other UAVs with reduced energy. In this paper, we propose a power allocation algorithm based on energy harvesting and energy cooperation to maximize the throughput of a UAV-assisted mmWave cellular network. Since there is channel-state uncertainty and the amount of harvested energy can be treated as a stochastic process, we propose an optimal multi-agent deep reinforcement learning algorithm (DRL) named Multi-Agent Deep Deterministic Policy Gradient (MADDPG) to solve the renewable energy resource allocation problem for throughput maximization. The simulation results show that the proposed algorithm outperforms the Random Power (RP), Maximal Power (MP) and value-based Deep Q-Learning (DQL) algorithms in terms of network throughput.

## 1. Introduction

Unmanned Aerial Vehicles (UAVs) are aircrafts without a human pilot on board. UAVs are able to establish on-demand wireless connectivity faster than terrestrial communications and they can adjust their height and position to provide robust channels with short-range line-of-sight links [1]. Therefore, UAV-aided wireless communication is a promising solution to provide temporary connections to devices without infrastructure coverage (e.g., due to severe shadowing in urban areas) or after telecommunication infrastructure has been damaged in natural disasters [1].

UAVs are identified as an important component of future-generation (5G/B5G) wireless networks due to their salient attributes (dynamic deployment ability, strong line-of-sight connection links and additional design degrees of freedom with the controlled mobility) [2].

In UAV-assisted wireless communications, UAVs are employed to provide wireless access for terrestrial users.

We distinguish between three use cases [1]:

(1) *UAV-aided ubiquitous coverage*: UAVs act as aerial base stations to achieve seamless coverage for a given geographical area. Some related applications are a fast communication service recovery in disaster scenarios and temporary traffic offloading in cellular hotspots.
(2) *UAV-aided relaying*: UAVs are employed as aerial relays between far-apart terrestrial users or user groups. Some examples of applications include UAV-enabled cellular coverage extension and emergency response.
(3) *UAV-aided information dissemination and data collection*: UAVs are used as aerial access points (APs) to disseminate (or collect) information to (from) ground nodes. Some related applications are UAV-aided wireless sensor networks and IoT communications.

The use of UAVs as aerial nodes to provide wireless sensing support has several advantages compared to ground sensing [3]. UAV-based sensing has a wider field of view due to the elevated height and reduced signal blockage of UAVs. In addition, UAV mobility enables to sense hard-to-reach poisonous or hazardous areas. Furthermore, the mobility of UAVs enables to perform sensing performance optimization by dynamically adjusting the trajectory of the UAVs.

UAV-based sensing has a wide range of potential applications, such as precision agriculture, smart logistics, 3D environment map construction, search and rescue, and military operations. There is a growing interest in the development of UAV-based sensing applications.

In Ref. [4], a complete framework for the data acquisition from wireless sensor nodes using a swarm of UAVs is introduced. It covers all the steps from the sensor clustering to the collision-avoidance strategy. In addition, a hybrid UAV-WSN system that improves the acquisition of environmental data in large areas has been proposed [5].

UAVs are a popular and cost-effective technology to capture high spatial and temporal resolution remote sensing (RS) images for a wide range of precision agriculture applications [6]. UAVs equipped with dual-band crop-growth sensors can achieve high-throughput acquisition of crop-growth information. IoT and UAV can monitor the incidence of crop diseases and pests from the ground micro and air macro perspectives, respectively [7]. In these applications, UAVs collect data from sensor nodes distributed over a large area. It is required to synchronize the UAV route with the activation period of each sensor node. The UAV path through all sensor nodes is optimized in Ref. [8] to reduce the flight time of the UAV and maximize the sensor nodes’ lifetime. In addition, an aerial-based data collection system based on the integration of IoT, LoRaWAN, and UAVs has been developed [9]. It consists of three main parts: (a) sensor nodes distributed throughout a farm; (b) a LoRaWAN-based communication network, which collects data from sensors and conveys them to the cloud; and (c) a path planning optimization technique for the UAV to collect data from all sensors.

In IoT communications [10], UAVs have also been proposed to assist the localisation of terrestrial Internet of Things (IoT) sensors and provide relay services in 6G networks. A mobile IoT device [11], located at a distant unknown location, has been traced using a group of UAVs equipped with received signal strength indicator (RSSI) sensors. In smart logistics, a UAV-based system aimed at automating inventory tasks has been designed and evaluated [12]. It is able to keep the traceability of industrial items attached to Radio-Frequency IDentification (RFID) tags.

In search and rescue operations, a real-time human detection and gesture recognition system based on a UAV with a camera is proposed [13]. The system is able to detect, track, and count people; it also recognizes human rescue gestures. Yolo3-tiny is used for human detection and a deep neural network is used for gesture recognition. UAV-assisted wireless networks can benefit from gigabit data transmissions by using 5G millimeter wave (mmWave) communications. The millimeter wave frequency band ranges from around 30 GHz to 300 GHz, corresponding to wavelengths from 10 to 1 mm. This key technology delivers higher data rates due to a higher bandwidth [14].

UAV-enabled mmWave networks offer a lot of potential advantages. On the one hand, the large available spectrum resources of mmWave communication and flexible beamforming can meet the requirements of high data rate and flexible coverage for UAVs serving as base stations (UAV-BSs) in UAV-assisted cellular networks [3]. These networks consist of a base station (BS) mounted on a flying UAV in the air, and mobile stations (MSs) distributed on the ground or at low altitude. High data rate communication links between the MSs and UAV BS are desirable in typical applications (e.g., to send control commands and large video monitoring traffic data from many camera sensors) [15]. On the other hand, the existence of a line-of-sight (LOS) path in the link from a UAV to the ground favors that mmWave communication obtains a high beamforming gain. However, higher propagation loss and the use of a large number of antennas in mmWave networks give rise to high energy consumption and UAVs are constrained by their low-capacity onboard battery.

Energy harvesting (EH) is a viable solution to reduce the energy cost of UAV-enabled mmWave networks; green energy can be harvested from renewable energy sources (e.g., solar, wind, electromagnetic radiations) to power UAVs. Energy-harvesting powered UAVs can prolong longer their operational duration as well as the wireless connectivity services they offer [16,17,18]. However, the random nature of renewable energy makes it challenging to maintain robust connectivity in UAV-assisted terrestrial cellular networks. Energy cooperation (also known as energy sharing or energy transfer) has been introduced in Ref. [19] to alleviate the harvested energy imbalance problem, where a source assists a relay by transferring a portion of its remaining energy to the relay.

We consider a UAV-assisted mmWave cellular network. Some UAVs will have plenty of energy because their flight duration is shorter or because they have harvested abundant energy due to better environmental conditions (e.g., sunshine without clouds). Energy cooperation allows that these UAVs can send their excessive energy to other UAVs with reduced energy.

In the literature, several works have investigated energy cooperation in renewable energy harvesting-enabled cellular networks. In Ref. [20], an adaptive traffic management and energy cooperation algorithm has been developed to jointly determine the amount of energy shared between BSs, the user association to BSs, and the sub-channel and power allocation in BSs. In Ref. [21], an energy-aware power allocation algorithm was developed in energy cooperation-enabled mmWave cellular networks. It uses renewable energy harvesting to maximize the network utility while keeping the data and energy queue lengths at a low level. In Ref. [22], a power allocation strategy is proposed that uses energy cooperation to maximize the throughput in ultra-dense Internet of Things (IoT) networks. However, these contributions do not analyze energy cooperation in UAV-assisted cellular networks.

Several authors proposed using energy transfer in UAV-enabled wireless communication systems. In Ref. [23], the total energy consumption of a UAV is minimized while accomplishing the minimal data transmission requests of the users; in the downlink the UAV transfers wireless energy to charge the users, while in the uplink the users utilize the harvested energy to transmit data to the UAV. Similarly, in Ref. [24], a downlink wireless power transfer and an uplink information transfer is proposed for mmWave UAV-to-ground networks. However, these contributions do not analyze energy cooperation between UAV-BSs.

In this paper, we propose a power allocation algorithm based on energy harvesting and energy cooperation to maximize the throughput of a UAV-assisted mmWave cellular network. This optimal power allocation and energy transfer problem can be regarded as a discrete-time Markov Decision Process (MDP) [25] with continuous state and action space. As statistical or complete knowledge about the environment, the real channel state, and the energy harvesting arrival is not easily observable, traditional model-based methods cannot be leveraged to tackle with this MDP. Therefore, we adopt multi-agent deep reinforcement learning (DRL) [26] to solve this problem and propose a multi-UAV power allocation and energy cooperation algorithm based on the Multi-Agent Deep Deterministic Policy Gradient (MADDPG) [27] method to optimize the policies for UAVs. To the best of our knowledge this is the first paper that analyses energy cooperation between UAV-BSs in UAV-assisted mmWave cellular networks and develops a DRL algorithm to maximize the network throughput. The proposed DRL algorithm can be applied in an emergency communication system for disaster scenarios. In these scenarios user devices that are out of the coverage range from UAVs cannot obtain wireless access. Therefore, it is important that UAVs increase their wireless coverage and reduce the channel access delay. Since UAVs are limited by their battery power, energy harvesting and energy cooperation are promising solutions to satisfy the requirements of an emergency communication system.

The contributions of this paper are summarized as follows:

- We study optimal power allocation strategies for UAV-assisted mmWave cellular networks when there is channel-state uncertainty and the amount of harvested energy can be treated as a stochastic process.
- We formulate the renewable energy resource allocation problem for throughput maximization using multi-agent DRL and propose an MADDPG-based multi-UAV power allocation algorithm based on energy cooperation to solve this problem.
- Simulation results show that our proposed algorithm outperforms the Random Power (RP), Maximal Power (MP) and value-based Deep Q-Learning (DQL) algorithms and achieves a higher average network throughput.

The paper is structured as follows. In Section 2, we analyze our system model. In Section 3, we state the renewable energy resource allocation problem and formulate it as an MDP with the objective to maximize the throughput. In Section 4, we introduce the MADDPG-based multi-UAV power allocation algorithm based on energy cooperation for solving the MDP. Simulation results are presented in Section 5. Finally, the paper is concluded in Section 6.

## 2. System Model

**Figure 1:**
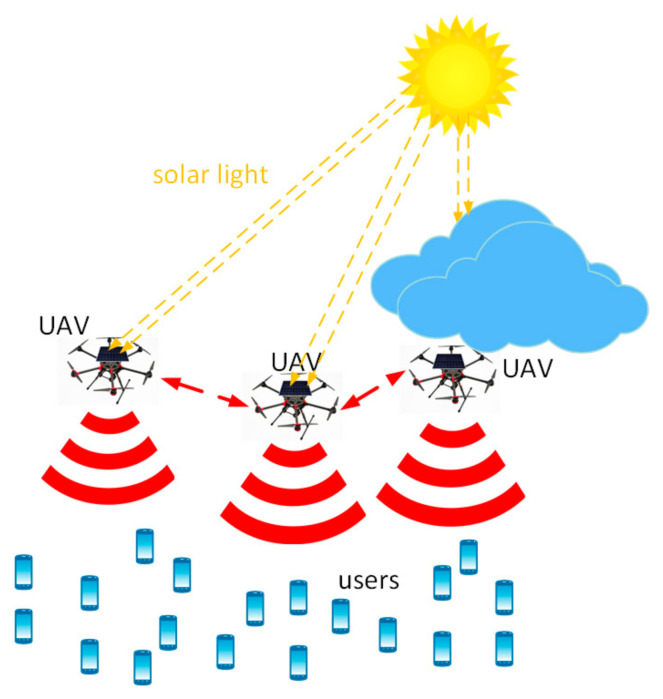
Network architecture.

Our network architecture is shown in Figure 1. For clarity, we summarize all the following notations and their definitions in Table 1. We consider a multi-antenna mmWave UAV-enabled wireless communication system, where multiple UAV-mounted aerial base stations (BSs) fly over the region and serve a group of users on the ground. We consider that each UAV is dedicated to serve a cluster of users with the same requirements. The locations of the UAV-enabled BSs are modelled as a Poisson point process (PPP) $\Phi_{j}$ with density $\lambda_{j}$. We consider that users are static. We assume that the location of users is modelled as a Poisson cluster process (PCP) $\Phi_{i}\;$ with density $\lambda_{i}\;$ [28]. We also assume that all UAVs are elevated at the same altitude $H_{j\;}\gg 0$.

UAVs are powered by hybrid energy sources. Onboard energy, along with a part of the harvested energy, is used to maintain the flight while the rest harvested energy is used to support the communication modules of the UAVs. The imbalance of energy harvesting between UAVs is compensated through energy cooperation.

The UAVs and user sets are denoted as $M=\left\{1,\ldots ,M\right\}$ and $L=\left\{1,\ldots ,L\right\}$. The total number of users served by UAV*j*, $j\in \left\{1,2,\ldots ,M\right\}$, can be represented by *L_j_* only associated with UAV*j*. For simplicity, the typical user set is associated with the closest UAV-BS; that is, the UAV that maximizes the average received SNR.

This paper focuses on the design of an optimal power allocation strategy to maximize the throughput for multi-UAV networks over *N* time slots. It is assumed that all UAVs communicate without the assistance of a central controller and have no global knowledge of wireless channel communication environments. This means that the channel state information (CSI) between a UAV and the mobile devices of the users is known locally. 

### 2.1. Blockage Model

A major challenge in mmWave communications is the blockage effect [14], namely, mmWave signals are blocked by physical obstacles in their propagation. We adopt the building blockage model introduced in Ref. [29], which defines an urban area as a set of buildings in a square grid. The mean number of buildings per square kilometer is ρ. The fraction of area covered by buildings to the total area is α. Each building has a height which is a Rayleigh-distributed random variable with scale parameter κ. The probability of a UAV having a line-of-sight (LOS) connection to the user i when the horizontal transmission distance is r is given by

(1) $$P^{L}\left(h_{t},h_{r},r\right)=\overset{\max\left(0,d-1\right)}{\underset{n=0}{\prod }}(1-\exp(-\frac{{\left(\max\left(h_{t},h_{r}\right)-\frac{\left(n+0.5\right)\left|h_{t}-h_{r}\right|}{d}\right)}^{2}}{2\kappa^{2}})$$

where$\;h_{t}$ is the transmitter height, $h_{r}\;$ is the receiver height, $d=\lfloor r\sqrt{\rho \alpha }\rfloor$ and ⌊.⌋ is the floor function. Furthermore, the probability for a non-line-of-sight (NLOS) transmission is $P^{N}\left(.\right)=\left(1-P^{L}\left(.\right)\right).$

### 2.2. UAV-to-Ground Channel Model

The path loss law in the UAV network is given by [30]:

(2) $$L\left(h_{t},h_{r},r\right)\frac{B\left(P^{L}\left(h_{t},h_{r},\;r\right)\right)C_{L}}{\sqrt{{\left(r^{2}+{\left|h_{t}-h_{r}\right|}^{2}\right)}^{\alpha_{L}}}}+\frac{B\left(P^{N}\left(h_{t},h_{r},\;r\right)\right)C_{N}}{\sqrt{{\left(r^{2}+{\left|h_{t}-h_{r}\right|}^{2}\right)}^{\alpha_{N}}}}$$

where $B\left(x\right)\;$ is a Bernoulli random variable with parameter x. The parameters $\alpha_{L}$ and $\alpha_{N}$ are the LOS and NLOS path loss exponents, and $C_{L}$ and $C_{N}$ are the intercepts of the LOS and NLOS links. 

The amplitude of the received UAV-to-ground mmWave signal can be modelled as a Nakagami-m fading distribution for both the LOS and NLOS propagation conditions at mmWave frequency bands. Let ${\hat{h}}_{S}$. be the small-scale fading term on the l-th link. ${\left|{\hat{h}}_{S}\right|}^{2}$ is a normalized Gamma random variable. ${\hat{h}}_{L}\sim \Gamma \left(N_{L},\frac{1}{N_{L}}\right)$ for LOS and ${\hat{h}}_{N}\sim \Gamma \left(N_{N},\frac{1}{N_{N}}\right)$ for NLOS, where $N_{L}$ and $N_{N}$ represent the Nakagami fading parameters for the LOS and NLOS links.

At time slot t, the LOS channel gain from the j-th UAV BS located at $x_{t}\;\in {\mathbb{R}}^{2}\;$to a i-th ground user located at $x_{r}\;\in {\mathbb{R}}^{2}\;$ can be expressed as

(3) $$H_{i,j}\left(h_{t},h_{r},\;x_{r}\right)\;=L\left(h_{t},h_{r},\;x_{r}\right)G_{x_{r}}{\left|{\hat{h}}_{x_{r}}\right|}^{2}$$

where $G_{x_{r}}$ is the directional antenna gain.

When the transmission has the maximum antenna gain, the channel gain is $H_{i,j}^{0}\left(h_{t},h_{r},\;x_{r}\right)\;=L(h_{t},h_{r},\;x_{r})G_{0}{\left|{\hat{h}}_{x_{r}}\right|}^{2}$.

### 2.3. Directional Beamforming

Beamforming, also known as spatial filtering, concentrates the signal energy over a narrow beam by means of highly directional signal transmission or reception; this way, the spectral efficiency is improved [14]. The narrow beams of the mmWave signals allow to achieve highly directional signals along the desired directions. We assume that $N_{B}$ and $N_{U}\;$antenna arrays are deployed at both the UAV-BSs and the mobile user sets, respectively. It is required to use efficient alignment policies (beam tracking, beam training, hierarchical beam codebook design, accurate estimation of the channel, etc.) to align the beams between transmitter and receiver [14].

**Figure 2:**
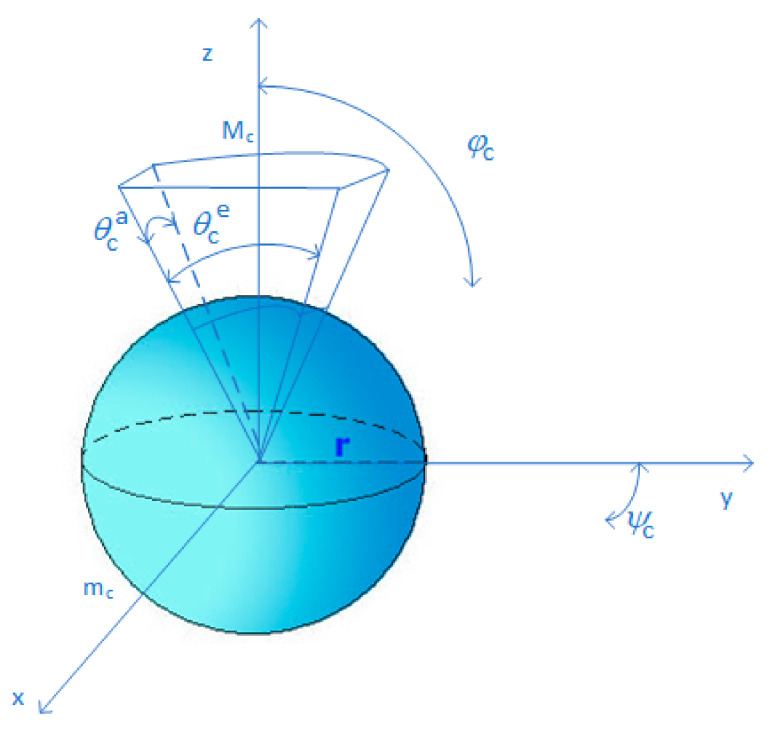
Sectorized antenna pattern.

We consider that the UAV-BS and user set antennas adopt a sectorized model that is shown in Figure 2. The antenna array pattern is characterized by four parameters: the half-power beamwidth in the azimuth plane $\theta_{c}^{a}$, the half-power beamwidth in the elevation plane $\theta_{c}^{e}$, the mean lobe gain $M_{c}\;$ and the side lobe gain $m_{c}$, where $c\in \left\{t,r\right\}\;$ refers to the transmitter (UAV-BS) and the receiver (user set), respectively.

The directivity gain at one receiver located at l from the j-th UAV-BS can be expressed as follows:

(4) $$G_{l}=G\left(M_{t},m_{t},\theta_{t}^{a},\theta_{t}^{e}\right)G\left(M_{r},m_{r},\theta_{r}^{a},\theta_{r}^{e}\right)$$

where $G(\theta_{c}^{a},\theta_{c}^{e}$,$\;M_{c},m_{c})$ denotes the directional antenna gain.

The transmitter and receiver should adjust their antenna directions towards each other to achieve the maximum beamforming gain $G_{0}=M_{t}M_{r}$.

### 2.4. Signal Model

The UAV-to-ground user pair communication is affected by the interference signals from the remaining UAVs. Therefore, the received signal-to-interference-plus-noise ratio (SINR) from UAVj located at $x_{t}\in {\mathbb{R}}^{2}\;$to user i at time slot t can be expressed as

(5) $$\gamma_{ij}\left(t\right)=\frac{H_{i,j}\left(t\right)P_{i,j}\left(t\right)}{I_{i,j}\left(t\right)+\sigma^{2}}$$

where $H_{i,j}\left(t\right)\;$ denotes the channel gain between UAV*j* and user i at time slot t, $P_{i,j}\left(t\right)\;$ is the transmit power selected by UAV*j* at time slot t, $I_{i,j}$ is the interference to UAV*j* that satisfies $I_{i,j}\left(t\right)=\underset{m\in M,\;m\neq j}{\sum }H_{i,m}\left(t\right)P_{i,m}\left(t\right)$ and $\sigma^{2}$ is the noise power level.

## 3. Problem Formulation

In this section, we investigate the optimal power allocation and energy transfer problem for throughput maximization in UAV-enabled mmWave networks, which can be regarded as MDP. Since the real channel state and energy harvesting arrival are not easily observable, traditional model-based methods are infeasible to tackle with this MDP. Therefore, we reformulate this problem using multi-agent DRL to make it solvable.

### 3.1. Throughput Maximization Problem

We introduce the following notation:

- $t\in \left\{1,2,\ldots ,N\right\}$ is one time slot of a finite horizon of *N* time slots.
- $\tau =t_{n}-t_{n-1},\;t\in \left\{1,2,\ldots ,N\right\}$ is the time slot duration.
- $E_{j}$ ∈ ℝ is the amount of harvested energy for UAV*j* at time slot t.
- C is the battery capacity of each UAV*j*.
- $B_{j}\in \left\{0,\ldots ,C\right\}$ is the battery state for UAV*j* at time slot t.
- $P_{j}$ is the transmission power allocated by UAV*j* to serve all its users.

The theoretical downlink rate of user $i,\;i\in L_{j}\;$ connected to UAV*j* at time slot t is given by

(6) $$R_{i,j}\left(t\right)=Wlog_{2}\left(1+\gamma_{i,j}\left(t\right)\right)$$

where W is the mm Wave transmission bandwidth.

The total throughput at timeslot t is

(7) $$U\left(t\right)=\overset{M}{\underset{j=1}{\sum }}\overset{L_{j}}{\underset{i=1}{\sum }}R_{i,j}\left(t\right)$$

The current battery capacity of each UAV*j* stores mainly the renewable energy harvested during the current time slot, the energy transferred by other UAVs during the energy cooperation process, and the remaining energy from the last time slot. 

The charging rate of the energy storage is usually less than the energy arrival rate, because of the limited energy conversion efficiency of the circuits. We consider that the charging rate and energy arrival rate are expressed as $E_{j}$ and $\hat{E_{j}}\;,\;$respectively. Therefore, $E_{j}=\eta \hat{E_{j}}$ ≥ 0, where 0< η≤1 is the imperfect conversion efficiency. In the rest of the paper, the energy arrival rate refers to the effective energy arrival rate that is assimilated by the system, i.e., the charging rate of the storage $E_{j}$.

After $E_{j}$ is harvested at time slot t, it is stored in the battery and is available for transmission in time slot t+1. The rechargeable battery is assumed to be ideal, which means that no energy is lost with energy storing or retrieving. Once the battery is full, the additional harvested energy is removed.

The battery energy level of UAV*j* at the time t+1 is

(8) $$B_{j}\left(t+1\right)=\min\{C,B_{j}\left(t\right)+E_{j}\left(t\right)-\tau P_{j}\left(t\right)-\overset{M}{\underset{j^{\prime }=1,j^{\prime }\neq j}{\sum }}\varepsilon_{jj^{\prime }}\left(t\right)+\overset{M}{\underset{j^{\prime }=1,j^{\prime }\neq j}{\sum }}\beta \varepsilon_{j^{\prime }j}\left(t\right)\}$$

where $\varepsilon_{jj^{\prime }}$ denotes the energy transferred from UAV*j* to UAV*j’*, $\varepsilon_{j^{\prime }j}$ denotes the energy transferred from UAV*j’* to UAV*j* and $\beta \in \left[0,\;1\right]$ is the energy transfer efficiency between two UAVs.

The problem can be formulated as follows:

**P1. Throughput optimization problem**

*Find:* $\;P_{i,j}\left(t\right)$

*Max:*

(9) $$\underset{t\in N}{\sum }U\left(t\right)$$

*Subject to:*

(10) $$0\leq E_{j}\left(t\right)\;\leq E_{max}\left(t\right)$$

(11) $$0\leq \tau P_{j}\left(t\right)\;\leq B_{j}\left(t+1\right)$$

(12) $$P_{j}\left(t\right)=\overset{L_{j}}{\underset{i=1}{\sum }}P_{i,j}\left(t\right)$$

(13) $$0\leq \overset{M}{\underset{j^{\prime }=1,j^{\prime }\neq j}{\sum }}\varepsilon_{jj^{\prime }}\left(t\right)\;\leq \min\{C,\;B_{j}\left(t\right)+E_{j}\left(t\right)\}$$

(14) $$\;0\leq \overset{M}{\underset{j=1,j\neq j^{\prime }}{\sum }}\varepsilon_{j^{\prime }j}\left(t\right)\;\leq \min\{C,B_{j^{\prime }}\left(t\right)+E_{j^{\prime }}\left(t\right)\}\;$$

The objective function of problem **P1** aims at finding the best values of $P_{i,j}\left(t\right)$ that maximizes the throughput. We observe that **P1** is a non-linear optimization problem.

Constraint (10) limits the harvested energy. Constraint (11) determines that the total energy consumed by each UAV should not exceed its battery level. Constraint (12) refers to the total allocated power by UAVj to serve all its users at time t. Since the energy storage at each UAV is limited, Constraint (13) expresses that the total energy transferred from UAV*j* to other UAVs should not exceed the current battery energy level. The same applies to Constraint (14) for the total transferred energy transferred from UAV*j’* to other UAVs.

The optimization problem may be solved only if the complete information about energy harvesting arrival and channel state information (CSI) is known. RL algorithms can achieve near-optimal performance even without prior knowledge about the CSI, the user arrival, the energy arrival, etc. [31]. In what follows, we analyze this problem under the MDP framework and reformulate it by adopting multi-agent RL.

### 3.2. Multi-Agent RL Formulation

The proposed problem can be considered an MDP. Therefore, multi-agent RL can be adopted to solve this problem efficiently.

Each UAV can be regarded as an agent in the proposed system and all the network settings can be regarded as the environment. UAVs can be characterized by a tuple $\langle S,\;{\left\{A_{j}\right\}}_{j\in M},P,{\left\{R_{j}\right\}}_{j\in \;M}\rangle$ as follows:

- S denotes the *state space* including all possible states of UAVs in the system at each time slot. The state of the j-th UAV, denoted by $s_{j}=(\gamma_{j}\left(t\right)$, $R_{j}\left(t-1\right),\;E_{j}\left(t\right)),\;$is described by the current SINR of the users served by UAV*j*, the link’s corresponding downlink rate $R_{j}\left(t-1\right)$ at the last time slot and the current harvested energy of UAV*j*, respectively.
- $\gamma_{j}\left(t\right)=\{\gamma_{1j}\left(t\right),\gamma_{2j}\left(t\right),\ldots \gamma_{L_{j}j}\left(t\right)\}$, $\forall j\in \left\{1,2,\ldots \;M\right\}$ refers to the SINR of the current serving users of UAV j. $R_{j}\left(t-1\right)=\{R_{1j}\left(t-1\right),R_{2j}\left(t-1\right),\ldots R_{L_{j}j}\left(t-1\right)$}, $\forall j\in \left\{1,2,\ldots \;M\right\}$ refers to the downlink rate of the current serving users of UAV j.
- $A_{j},j\in M$ denotes the *action space* consisting on all the available actions of j-th UAV at each time slot. The action of the j-th UAV, denoted by $a_{j}$, is defined as $a_{j}=\left(P_{j}\right)$. This means that each UAV selects the power allocated to serve its users. At state $s_{j}$, the available action set of the j-th UAV is expressed as $A_{j}=A\left(s_{j}\right)$.
- $P:S^{M}\times \prod_{j=1}^{M}A_{j}\to \prod \left(S\right)$ is the *state transition function*, which maps the state spaces and the action spaces of all UAVs in the current time slot to their state spaces in the next time slot.
- $R_{j},j\in \;M\;$is the *reward function* of the j-th UAV, which maps the state spaces and the action spaces of the UAV in the current time slot to its expected reward. The reward of the j-th UAV, denoted by $r_{j}$, is defined as $r_{j}=U\left(t\right)$.

Each UAV is motivated to maximize the throughput by making decisions on power allocation. In our system, the *policy* of a UAV is defined as a mapping from its state space to its action space, denoted by π. At the beginning of each time slot the j-th UAV observes the state of all UAVs$,\;\;s=\left\{s_{j}\right\}$, from state space $S^{M}$, and takes an action $a_{j}$ based on its policy $\pi_{j}$. Actually, a UAV cannot know the states of other UAVs by itself. However, before making decision in a cycle, the UAV can observe the states of other UAVs by sending a beacon. After making the decision, the UAV will keep its decision unchanged till the end of the current cycle. The policy of the j-th UAV can be defined as $a_{j}=\pi_{j}\left(s\right)$, where s is the state of all UAVs in the system and $a_{j}\;$is the action of the j-th UAV. After that, the UAV receives a reward $r_{j}$ and then observes the next state $s^{\prime }$, namely, the states of all UAVs at the beginning of the next time slot. Therefore, the throughput maximization problem can be transformed into maximizing the total accumulated rewards of all UAVs in the system by optimizing their policies; that is,

**P2. Maximization of the total accumulated rewards**

*Max:*

(15) $$\underset{t\in N}{\sum }r_{j}$$

## 4. Proposed Multi-Agent Reinforcement Learning Algorithm

Our target is to design an efficient algorithm for power allocation and energy transfer for UAVs that maximizes the throughput. Existing approaches such as dynamic programming is not suitable for such challenging tasks. Therefore, we adopt an RL algorithm to cope with the problem (P2). DRL has a better performance on tasks that have a sophisticated state space and time-varying environment than traditional reinforcement learning. There are different kinds of DRL that could deal with different situations, e.g., Deep Q-Learning (DQL) could work well with a limited action space and deep deterministic policy gradient (DDPG [32]) has a remarkable performance with continued action space. There are two ways to apply the DDPG algorithm in our proposed scenario. The first solution would be to have a global DDPG agent that outputs all UAVs’ actions and there is only one reward function in this centralized fully observable case. However, if we consider one action (transmission power) with an infinite action space for each UAV, a global agent would have to cope with an exponential number of actions, which would become a problem. Another solution is to apply DDPG on each UAV; in this case, we would have multiple DDPG agents that output actions for each UAV. However, this solution is inefficient compared to MADDPG [27] because at every time slot each UAV agent will be trying to learn to predict the actions of the other UAVs while also taking its own actions. On the other hand, MADDPG is the state-of-the art solution for multi-agent DRL. It employs a centralized critic and decentralized actors. Actors can use the estimated policies of other agents for learning. This way, agents are supplied with information about the other UAVs’ observations and potential actions, transforming an unpredictable environment into a predictable one. This additional information is used to simplify the training, as long as it is not used at the test time (centralized training with decentralized execution). In this paper, we propose a MADDPG-based multi-UAV design algorithm for power allocation and energy transfer to optimize multiple UAVs’ policies. Afterwards, we introduce the training process of the proposed algorithm.

### 4.1. Algorithm Design

MADDPG is an actor-critic algorithm [33] designed for multi-agent environments. Actors are responsible for learning policies and critics evaluate the actors’ action choices.

MADDPG adopts a strategy based on centralized training and distributed execution. Each UAV works as an agent and has an actor network $\theta_{j}^{\mu }\in \left\{\theta_{1}^{\mu },\;\ldots ,\theta_{M}^{\mu }\right\}$, which means that each agent j takes continuous policies $\mu_{j}$ with regard to parameters $\theta_{j}^{\mu }$. Each agent also has a critic network $\theta_{j}^{Q}\in \left\{\theta_{1}^{Q},\;\ldots ,\theta_{M}^{Q}\right\}$. The critics are fed with information about the global state S and actions A of all agents. They are aware of the actions of all agents and output a Q value that describes how good joint action A is on state S. Target networks serve as stable targets for learning. Each agent has an actor target network ${\theta_{j}^{\mu }}^{\prime }\in \left\{{\theta_{1}^{\mu }}^{\prime },\;\ldots ,{\theta_{M}^{\mu }}^{\prime }\right\}$ and a critic target network ${\theta_{j}^{Q}}^{\prime }\in \left\{{\theta_{1}^{Q}}^{\prime },\;\ldots ,{\theta_{M}^{Q}}^{\prime }\right\}$.

The loss function of the critic network is calculated by

(16) $$L\left(\theta_{j}^{Q}\right)=E_{s^{t}\sim D}[{\left(Q_{j}(s^{t},a^{t}|\theta_{j}^{Q})-y_{j}^{t}\right)}^{2}]$$

where D is the replay buffer that stores historical experience. $y_{j}^{t}$ is defined as

(17) $$y_{j}^{t}=r_{j}^{t}+\lambda Q_{j}^{\prime }\left(s^{t+1},a_{j}^{t+1}{}^{\prime }|\theta_{j}^{Q}{}^{\prime }\right){|}_{{a_{j}^{t+1}}^{\prime }=\mu_{j}^{\prime }\left(s_{j}^{t+1}\right)}$$

The gradient of the expected reward for agent j with deterministic policies $\mu_{j}\;$is given by

(18) $$\nabla_{\Theta_{j}^{\mu }}\;J(\Theta_{j}^{\mu })=E_{s^{t}\sim D}[\nabla_{a_{j}^{t}}\;Q_{j}\left(s^{t},a_{j}^{t}\right)\nabla_{\theta_{j}^{\mu }}a_{j}^{t}{|}_{a_{j}^{t}=\mu_{j}\left(s_{j}^{t}\right)}]$$

The MADDPG algorithm is shown as Algorithm 1.
**Algorithm 1:** MADDPG-based multi-UAV power allocation algorithm based on energy cooperation
**Input**: The structures of the actor network, critic network, and their target networks; Number of episodes **Output**: Policy
$\pi_{j}$; 
1: Initialize the replay memory
RM with size *X*. 
2: Initialize critics
$Q_{j}(s^{t},a^{t}|\theta_{j}^{Q})$ and actors $\mu_{j}(s_{j}|\;\theta_{j}^{\mu })$ with random weights $\theta_{j}^{Q}$$\;\mathrm{and}\;{\theta_{j}^{\mu }}^{\prime }$

Initialize target networks with random weights
${\theta_{j}^{Q}}^{\prime }$ and ${\theta_{j}^{\mu }}^{\prime }$
3: Receive initial state
$s_{j}^{t}$; 
4: **for**
$\;n_{epi}=1:N_{epi}$ **do**

5:  **for**
 t=1: N do 
6:   Each agent selects an action
$a_{j}^{t}=$$\mu_{j}(s_{j}^{t}|\;\theta_{j}^{\mu })$$+\;v_{j}$$,\;\mathrm{where}\;v_{j}$ is the exploration of the action.
7:   Receive reward
$r_{j}^{t}$ and observe next state $s_{j}^{t+1}$
8:   Store
$(s_{j}^{t}$$,\;a_{j}^{t}$$,\;r_{j}^{t},s_{j}^{t+1})$ in the replay memory RM
9:   **if**
 RM is full **do**

10:    Sample a batch of random samples
$((s_{j}^{t}$$,\;a_{j}^{t}$$,\;r_{j}^{t},s_{j}^{t+1})$ from RM. 
11:    Set with (17). 
12:    Update the actor of the estimated network
$\theta_{j}^{\mu }\;$with (18). 
13:    Update the critic of the estimated network
$\theta_{j}^{Q}\;$ with (16).
Update the target network parameters with
${\theta_{j}^{Q}}^{\prime }\leftarrow \xi \theta_{j}^{Q}+\left(1-\xi \right){\theta_{j}^{Q}}^{\prime }$$,\;{\theta_{j}^{\mu }}^{\prime }\leftarrow \xi \theta_{j}^{\mu }+\left(1-\xi \right){\theta_{j}^{\mu }}^{\prime }$
14:  **end for**

15: **end for**

16: Return
$\theta_{j}^{\mu }$. 
17: Choose optimal action
$a_{j}^{t}*=$
$\mu_{j}(s_{j}^{t}|\;\theta_{j}^{\mu })$ at time t.

### 4.2. Complexity Analysis

The computation complexity and the space complexity for the proposed MADDPG algorithm can be estimated by the replay memory and the neural networks’ architecture. In MADDPG, the training network of each agent consists of two sets of actor networks and two sets of critic networks. The time complexity (computations) is given with regard to the floating-point operations per second (FLOPS). The neural networks are fully connected layer networks. For dot products of a P vector and a P×Q matrix, the FLOPS is $\left(2P-1\right)Q$ because for every column in matrix we need to multiply P times and add $\left(P-1\right)$ times. It is also necessary to derive the computations of the activation layers. In this case addition, subtraction, multiplication, division, exponentiation, square root, etc., count as a single FLOP. Therefore, the computations are Q with Q inputs for ReLU layers, 4×Q for sigmoid layers and 6×Q for tanh layers.

We consider that $u_{a,j}$ is the unit number in the j-th layer of the actor, and $u_{c,j}$ the number of neurons in the k-th layer of the critic. The number of layers for the actor and critic networks are J and K, respectively.

Therefore, the time complexity of the training is:

(19) $$v_{act}u_{i}+2\overset{J-1}{\underset{j=0}{\sum }}u_{a,j}u_{a,j+1}+2\overset{K-1}{\underset{k=0}{\sum }}u_{c,k}u_{c,k+1}\;\;=O\left(\overset{J-1}{\underset{j=0}{\sum }}u_{a,j}u_{a,j+1}+\overset{K-1}{\underset{k=0}{\sum }}u_{c,k}u_{c,k+1}\right)\;$$

where $u_{i}$ means the unit number in the i-th layer and $v_{act}$ means the corresponding parameters determined by the type of the activation layer.

Space is needed to store the learning transition. The memory replay in MADDPG occupies some space to store the state sets; therefore, the space complexity is N. For a fully connected layer in both the actor and the critic network, there is a P×Q matrix and a Q bias vector. The memory for a fully connected layer is $\left(P+1\right)Q.\;$The space complexity of the neural networks is given by

(20) $$2\overset{J-1}{\underset{j=0}{\sum }}u_{a,j}u_{a,j+1}+2\overset{K-1}{\underset{k=0}{\sum }}u_{c,k}u_{c,k+1}+v_{act}u_{i}+N\;\;=O\left(\overset{J-1}{\underset{j=0}{\sum }}u_{a,j}u_{a,j+1}+\overset{K-1}{\underset{k=0}{\sum }}u_{c,k}u_{c,k+1}\right)+O\left(N\right)\;$$

## 5. Simulation Results

In this section, we present the simulation results of the multi-UAV power allocation and energy transfer algorithm. The simulation parameters are given in Table 2. We assume that the mmWave network is operated at 28 GHz. We set each actor or critic network as a four-layer neural network with two hidden layers, in which the number of neurons in the two hidden layers are 64 and 128, respectively. The activation function for the hidden layers is rectified linear unit (ReLU) $f\left(x\right)=\max\left(o,x\right)$. The Adam algorithm is adopted as the optimizer and the learning rate is set as exponentially decayed to improve the performance of the training.

**Figure 3:**
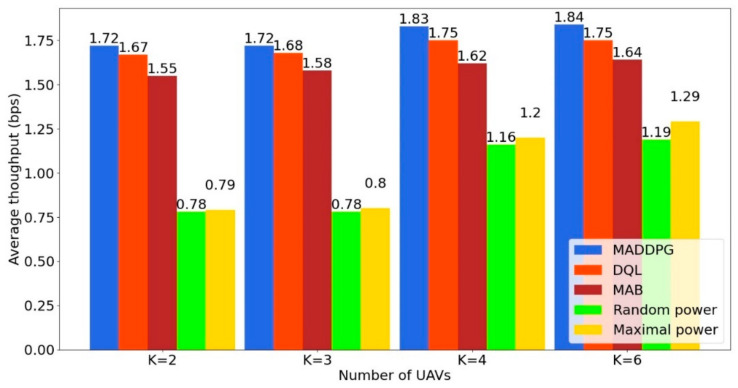
Average throughput as a function of the number of UAVs.

Next, we compare the proposed algorithm MADDPG with the Random Power (RP), Maximal Power (MP), Multi-Armed Bandit (MAB) and value-based Deep Q-Learning (DQL) algorithms. Upper confidence bound (UCB) is used to solve the MAB problem. RP and MP are two classical algorithms, whereas MADDPG, DQL and MAB are RL-based algorithms. Maximal Power (MP) consumes as much energy as possible in each time slot to improve its immediate throughput regardless of the future and the performance of the other UAVs. Random Power (RP) only consumes a part of the energy chosen randomly in each time slot. The average throughput for the five power allocation schemes is shown in Figure 3 as a function of the number of UAVs. We observe that the average throughput is increased with the number of UAVs. MADDPG achieves the highest average throughput in all testing scenarios and outperforms the other algorithms. The gap between the RP/MP allocation schemes and the rest of the algorithms is decreased when the number of UAVs increases.

**Figure 4:**
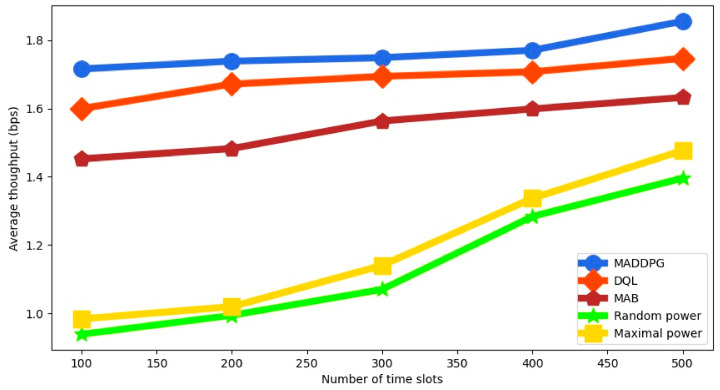
Average throughput as a function of the number of time slots.

The average throughput for the different policies is shown in Figure 4 as a function of the number of time slots. We can observe that MADDPG always outperforms the other algorithms. Since MADDPG does not divide the action space into discrete values like DQL, it can select a better action in each time slot without quantization errors. We notice that when the number of time slots increases, the average throughput is much larger for the RL-based algorithms, because they can adjust the transmission power in a smart way. MAB shows similar but worse behavior than DQL.

**Figure 5:**
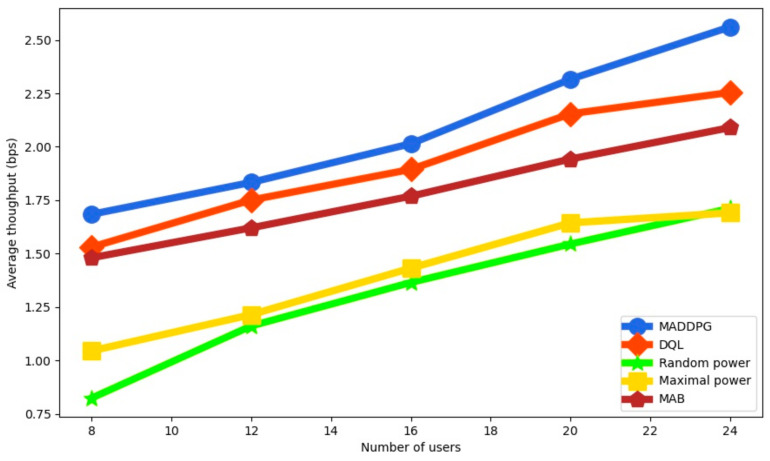
Average throughput as a function of the number of users with 4 UAVs.

The average throughput as a function of the number of users is illustrated in Figure 5 with four UAVs. We observe that the average throughput is increased because more users are served. MADDPG improves the average throughput compared to the other approaches. It is 13.6% higher than DQL, 22.53% higher than MAB, 46.24% higher than RP and 49.56% higher than RP for 24 users, which proves the effectiveness of the proposed approach.

**Figure 6:**
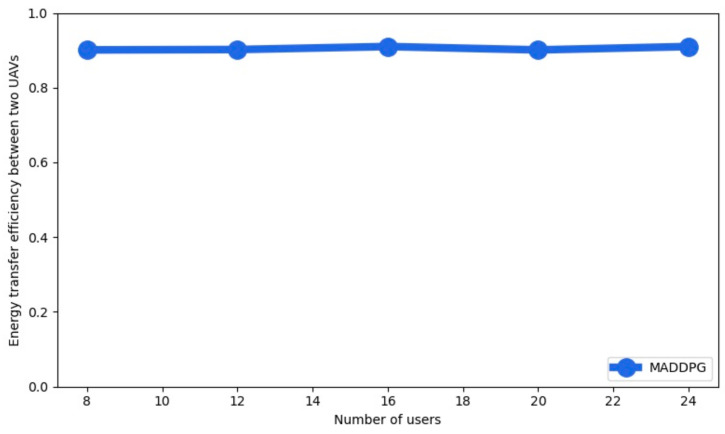
Energy transfer efficiency as a function of the number of users.

The energy transfer efficiency between two UAVs is shown in Figure 6 as a function of the number of users with four UAVs for MADDPG. We observe that the energy transfer efficiency has a high value for a different number of users.

**Figure 7:**
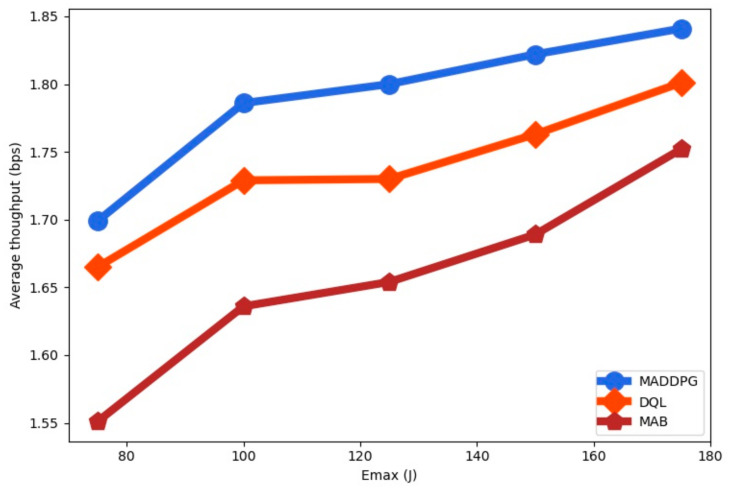
Average throughput as a function of the energy arrival $E_{max}$.

The average throughput for the RL-algorithms is shown in Figure 7 as a function of the energy arrival $E_{max}$. We observe that the average throughput is increased with the maximum energy harvested $E_{max}$. The average throughput for MADDPG is higher than for DQL and for MAB. Since the amount of collected energy is lower than the size of the battery capacity the throughput is increased for larger values of $E_{max}$.

**Figure 8:**
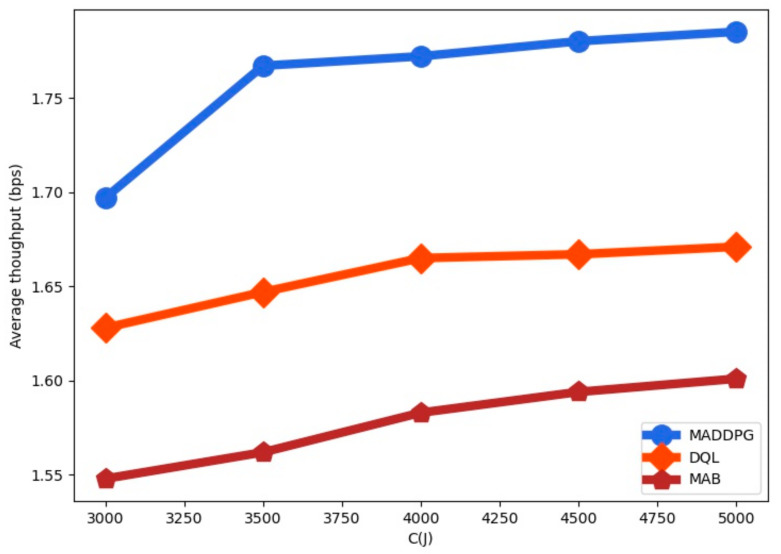
Average throughput as a function of the battery capacity C.

The average throughput for the RL-algorithms is shown in Figure 8 as a function of the battery capacity C. We observe that the average throughput is increased with the battery capacity. The average throughput is higher for MADDPG than for DQL and for MAB. We observe that when the battery capacity is increased the throughput values for the policies tend to stabilize since the value of $E_{max}$ limits the system throughput increase.

**Figure 9:**
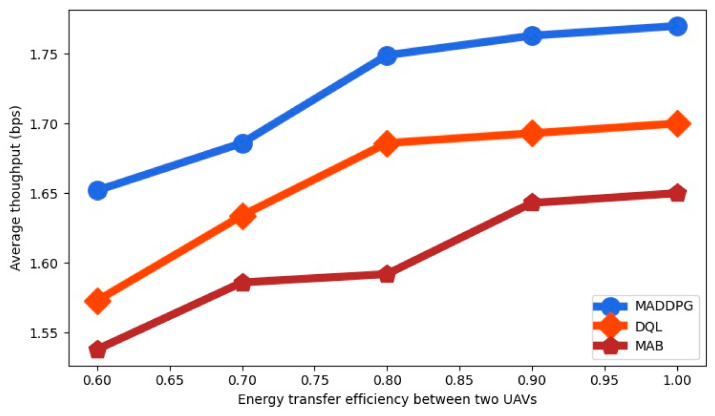
Average throughput as a function of the energy transfer efficiency between two UAVs.

The average throughput for the RL-algorithms is shown in Figure 9 as a function of the energy transfer efficiency between two UAVs β. We observe that the average throughput is increased with the energy transfer efficiency. The average throughput is higher for MADDPG than for DQL and for MAB for all the values of β.

**Figure 10:**
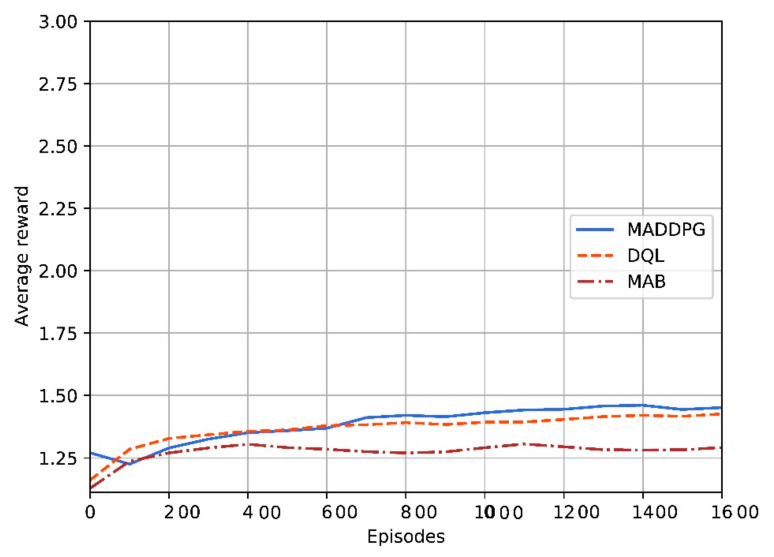
Convergence behavior for the reinforcement learning protocols.

The convergence behavior of the reinforcement-based algorithms in terms of average reward is shown in Figure 10 for a network of three UAVs and 14 users. The convergence behavior is around 1400 iterations for MADDPG. For DQL it is shorter (around 600 iterations). Finally, for MAB the convergence time is larger (around 1100 iterations).

## 6. Conclusions

In this paper, the optimal power allocation strategies for UAV-assisted mmWave cellular networks were analyzed. A power allocation algorithm based on energy harvesting and energy cooperation is proposed to maximize the throughput of a UAV-assisted mmWave cellular network. Since there is channel-state uncertainty and the amount of harvested energy can be treated as a stochastic process, we propose an optimal multi-agent deep reinforcement learning algorithm (DRL) named Multi-Agent Deep Deterministic Policy Gradient (MADDPG) to solve the renewable energy resource allocation problem for throughput maximization. The simulation results show that the proposed algorithm outperforms the Random Power (RP), Maximal Power (MP), Multi-Armed Bandit (MAB) and value-based Deep Q-Learning (DQL) algorithms in terms of network throughput. Besides, the RL-based algorithms outperform the traditional RP and MP algorithms and show improved generalization performance, since they can adjust the transmission power in a smart way. The average throughput is increased with the number of UAVs, the energy arrival, the battery capacity and the energy transfer efficiency between two UAVs. When the battery capacity is increased the throughput values for the RL policies tend to stabilize since the value of $E_{max}$ limits the system throughput increase.

MADDPG can be applied to many tasks with discrete or continuous state/action space and joint optimization problems of multiple variables. It can successfully solve user scheduling, channel management and power allocation problems in different types of communication networks. The optimization of the locations of UAVs and their trajectories [34] is an important topic. Therefore, we will further investigate the development of a joint power allocation and UAV trajectory approach as future work.

## Figures and Tables

**Table 1:** List of notations.

Notations	Definitions
M	Number of UAVs
L	Number of user sets
ρ	Mean number of buildings per square kilometer
κ	Scale parameter
α	Fraction of area covered by buildings to the total area
$L\left(t\right)$	Path loss
$\alpha_{L}$	LOS path loss exponent
$\alpha_{N}$	NLOS path loss exponent
$C_{L}$	Intercept of the LOS link
$C_{N}$	Intercept of the NLOS link
${\hat{h}}_{S}$	Small-scale fading
$N_{L}$	Nakagami fading parameter for LOS link
$N_{N}$	Nakagami fading parameter for NLOS link
$H_{i,j}\left(t\right)$	Channel gain from the *j*-th UAV BS to a *i*-th ground user
$G_{x_{r}}$	Directional antenna gain
$G_{0}$	Maximum antenna gain
$\theta_{c}^{a}$	Azimuth plane
$\theta_{c}^{e}$	Elevation plane
$M_{c}$	Mean lobe gain
$m_{c}$	Side lobe gain
$\gamma_{ij}\left(t\right)$	Signal-to-interference-plus-noise ratio from UAV*j* to user *i*
$P_{i,j}\left(t\right)$	Transmit power selected by UAV*j*
$P_{max}$	Maximum transmission power
$I_{i,j}$	Interference to UAV*j*
$\sigma^{2}$	Noise power level
N	Total number of time slots
τ	Te slot duration
$E_{j}$	Amount of harvested energy for UAV*j*
$E_{max}$	Maximum harvested energy
C	Battery capacity of each UAV*j*
$B_{j}$	Battery state for UAV*j*
$R_{i,j}\left(t\right)$	Downlink rate of user i
W	MmWave transmission bandwidth
$U\left(t\right)$	Total throughput
${}_{jj^{\prime }\;}$	Energy transferred from UAV*j* to UAV*j’*
β	Energy transfer efficiency between two UAVs.
S	State space
$A_{j}$	Action space
P	State transition function
$R_{j}$	Reward function

**Table 2:** Simulation parameters.

Parameters	Values	
Number of UAVs	4	
Maximum flying altitude of UAVs	100 m	
Number of users	12	
Mean number of buildings per square kilometer is ρ	300/km^2^	
Fraction of area covered by buildings to the total area α.	0.5	
Scale parameter κ	20 m	
$\mathrm{LOS}\;\mathrm{intercept}\;C_{L}$	1.39	
$\mathrm{NLOS}\;\mathrm{intercept}\;C_{N}$	1.39	
$\mathrm{LOS}\;\mathrm{path}\;\mathrm{loss}\;\mathrm{exponent}\;\alpha_{L}$	2	
$\mathrm{NLOS}\;\mathrm{path}\;\mathrm{loss}\;\mathrm{exponent}\;\alpha_{N}$	3	
$\mathrm{LOS}\;\mathrm{Nakagami}\;\mathrm{fading}\;\mathrm{parameter}\;N_{L}$	3	
$\mathrm{NLOS}\;\mathrm{Nakagami}\;\mathrm{fading}\;\mathrm{parameter}\;N_{N}$	2	
Available bandwidth W	1 GHz	
$\mathrm{Noise}\;\mathrm{figure}\;N_{F}$	10 dB	
$\mathrm{Noise}\;\mathrm{power}\;\sigma^{2}$	$-170+10log_{10}(W$$)+N_{F}$ dBm = −70 dBm	
$\mathrm{Transmission}\;\mathrm{power}\;P_{max}$	(0,20) dBm	
Battery capacity *C*	4000 J	
$\mathrm{Energy}\;\mathrm{Arrival}\;E_{max}$	(0,125) J	
Energy transfer efficiency between two UAVs β	0.9	
Number of episodes	5000	
Number of time slots per episode	500	
Batch size	500	
Replay memory size	50,000	
Learning rate for DQL	$10^{-3}$	
Learning rate for MADDPG	Actor	Critic
	$10^{-4}$	$10^{-3}$
